# Experiences and Practices in the Current Prevention and Control of the Novel Coronavirus Pneumonia in China

**DOI:** 10.1017/dmp.2020.173

**Published:** 2020-05-29

**Authors:** Shen Shao, Zichen Zhou, Yue Li, Shuyu Liu, Lu Lu, Shike Hou, Bin Fan, Chunxia Cao, Haojun Fan

**Affiliations:** Institute of Disaster Medicine, Tianjin University, China

**Keywords:** experience and practice, health emergency management, novel coronavirus pneumonia

## Abstract

Since December 2019, several new infectious diseases, mainly lung diseases caused by novel coronavirus infections, have been discovered in Wuhan, Hubei Province. With the spread of the epidemic, cases in other regions of China and abroad have been confirmed. This sudden outbreak of a new type of infectious disease has seriously threatened people’s health and safety, and China has adopted strong prevention and control measures in response. To provide a reference for international health emergency management workers, this article summarizes, from an academic perspective, the main prevention and control measures taken in China.

Since December 2019, hospitals in Wuhan, Hubei Province, have successively identified multiple cases of unexplained pneumonia appearing to correspond to exposure to the Huanan Seafood Market in Wuhan. These cases were confirmed as acute respiratory infectious diseases caused by novel coronavirus (2019-nCoV). Subsequently, the novel coronavirus pneumonia (coronavirus disease 2019, COVID-19) spread rapidly throughout the country, especially in Hubei Province and Wuhan. On January 31, 2020, the World Health Organization (WHO) designated this epidemic as a “Public Health Emergency of International Concern” (PHEIC).^1^ In response, the Central Committee of the Communist Party of China (CPC) and the State Council of China, recognizing the great importance of timely prevention and control, adopted various rescue and emergency safeguard measures to curb the spread of the epidemic in China.

This article mainly examines early work done to prevent and control COVID-19 from an academic perspective. While reflecting on China’s institutional advantages for epidemic prevention and control, it also outlines challenges that China may face regarding COVID-19 control in the future. We also aim to explore the implications of such epidemic prevention and control for future medical emergency management with a view that integrates theory and practice.

## BASIC SITUATION OF COVID-19 IN 2019

### Etiology

The2019-nCoV belongs to the β-single-stranded RNA virus. It is enveloped, its diameter is 60-140 nm, its genetic material contains 26-32 bases, and it has 85% homology with the virus that causes severe acute respiratory syndrome (SARS).^2^ The virus is resistant to ultraviolet radiation and heat. Exposure to 56°C for 30 min and lipid solvents such as ether, 75% ethanol, chlorine-containing disinfectant, peracetic acid, and chloroform can effectively inactivate the virus.^2^

### Clinical Characteristics

Based on the current epidemiological survey in China, the incubation period of COVID-19 is 1-14 days. The main manifestations are fever, fatigue, and dry cough. Some patients have symptoms such as nasal congestion, runny nose, sore throat, and diarrhea. Mild cases can manifest as low fever and slight fatigue, with no manifestations of pneumonia. Severe and critical patients will have moderate-to-low fever during the course of the disease or no obvious fever at all.

### Epidemic Overview

The case definitions of confirmed cases and suspected cases were diagnosed according to the diagnostic and treatment guidelines for COVID-19. Confirmed cases are diagnosed with 1 of the etiological or serological evidences. Suspected persons are diagnosed according to epidemiological history and clinical manifestations.^2^ As of 24:00 on March 6, China had 80,651 confirmed cases, a total of 55,404 discharged patients, 3070 deaths, and 502 existing suspected cases. A total of 672,458 instances of close contact had been tracked, and 26,730 close-contact patients were still under medical observation.^3^ A total of 98,192 confirmed cases were reported abroad; 3380 persons died.

### Epidemiological Characteristics

Current research shows that the virus that causes COVID-19 shares more than 85% homology with bat SARS-like coronaviruses (bat-SL-CoVZC45).^2,4^ The sources of infection currently seen are mainly patients with 2019-nCoV infection. Asymptomatic infection may also become a source of infection.^5^

Transmission of the virus happens mainly through respiratory droplets and close contact. There is the possibility of aerosol transmission in a relatively closed environment for a long-time exposure to high concentrations of aerosol. As the novel coronavirus can be isolated in feces and urine, attention should be paid to feces or urine contaminated environmental that leads to aerosol or contact transmission.^2^

At the beginning of the epidemic, all population types were generally susceptible to 2019-nCoV.^6^

## EMERGENCY MANAGEMENT MEASURES IN RESPONSE TO THE EPIDEMIC

Since the SARS crisis in 2003, China has established a “1 case, 3 systems” framework for health emergency management, which includes systems, mechanisms, legal systems, and plans. In 2018, the Ministry of Emergency Management of China was established to be mainly responsible for natural disasters and accidental disasters. Public health emergencies are also overseen by the National Health Commission of the People’s Republic of China (NHC of China).

In the emergency management of the present epidemic, the Chinese government has shown high responsibility regarding people’s safety and has adopted timely and effective preventive and control measures. Many of these measures have far exceeded the requirements of the International Health Regulations.

**FIGURE 1:**
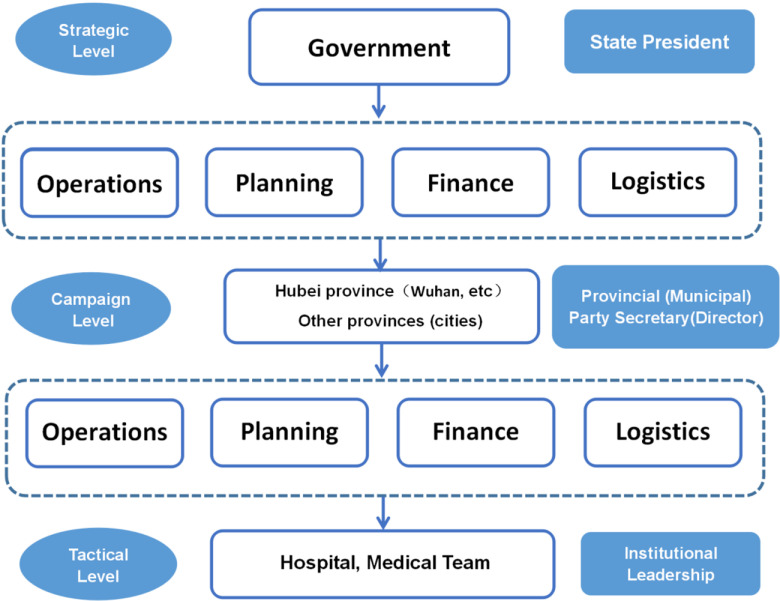
Three-Level Command System.

A 3-level command system is used. The first level is the strategic level, which is composed of the Central Committee of the CPC, the State Council of China, and the Central Military Commission. This level uniformly controls national prevention and the control work. The second level is the campaign level. The provincial (municipal) party secretary or provincial (municipal) director serves as the commander to carry out emergency management work in the province (municipality) and coordinate external reinforcements. The third level is the tactical level, where the commander of the hospital and the foreign-aid medical team are in charge (see Figure 1).

### Strategic Level of Emergency Management

#### Unified Command and Coordination

The Central Committee of the CPC set up a leading group for the response to the epidemic. The general secretary personally deployed it and sent a steering group to areas with severe outbreaks, such as Hubei Province. The government established a working mechanism for the joint prevention and control of the epidemic,^7^ including epidemic prevention and control, medical treatment and medical emergency supplies, security, transportation, and media. The government has also improved the military-ground cooperation mechanism to ensure that various prevention and control measures are enacted in the right places.

#### Coordinating Superior National Medical and Nursing Forces to Strengthen Medical Treatment in Hubei Province

Standardized treatment: The NHC of China has continuously revised the diagnosis and treatment plan; the updated version is the ‘‘Novel Coronavirus Infected Pneumonia Diagnosis and Treatment Plan” (version 7). It has become the unified guide for the diagnosis and treatment of COVID-19 in China.

Strengthening treatment: Based on medical resources in Hubei Province, the government has coordinated local and military medical resources and mobilized more than 40,000 medical personnel from across the country to support Wuhan. The government established a 1-to-1 support relationship between 16 provinces to support cities in Hubei Province and coordinated their strengths to treat patients.

Expert teams have been sent to Wuhan many times. Dr. Zhong Nanshan, Dr. Gaofu, and other scholars visited Wuhan to investigate and confirm the source and transmission characteristics of 2019-nCoV.

#### Guarantee the Production and Supply of Emergency Resources

The material security team implements the unified management and allocation of protective medical clothing, such as N95 masks and medical goggles, as well as negative pressure ambulances and related drugs in accordance with the needs of areas where materials are scarce.

The NHC of China developed the “Online Declaration System for Emergency Materials for Emergency Response and Disposal,” which summarizes emergency material needs for the prevention and control of the epidemic. The Ministry of Industry and Information Technology has developed a national key material security platform that counts and dispatches the production capacity, output, and inventory of material enterprises, focusing on guaranteeing the deployment of medical supplies in key epidemic areas in Wuhan.

As of 24:00 on March 5, the material security team has coordinated domestic production enterprises and has sent 162,180 protective clothing items, 348,770 N95 masks, and 200,000 Medical gloves^8^ to Hubei Province.

#### Epidemic Prevention and Management

On January 20, 2020, the NHC of China included COVID-19 in the Class B infectious diseases specified in the Infectious Disease Law of China and adopted preventive control measures for Class A infectious disease.^9^

Those who deliberately spread the pathogen of an infectious disease, who conceal their contact history with an epidemic area, or refuse to accept quarantine will be convicted and punished for crimes against public safety. The above measures are intended to ensure social stability and order.

### Emergency Management Measures in Hubei Province

As of March 6, there were 22,177 confirmed cases nationwide and 21,239 confirmed cases in Hubei, of which 19,011 were in Wuhan alone.^10^

**FIGURE 2:**
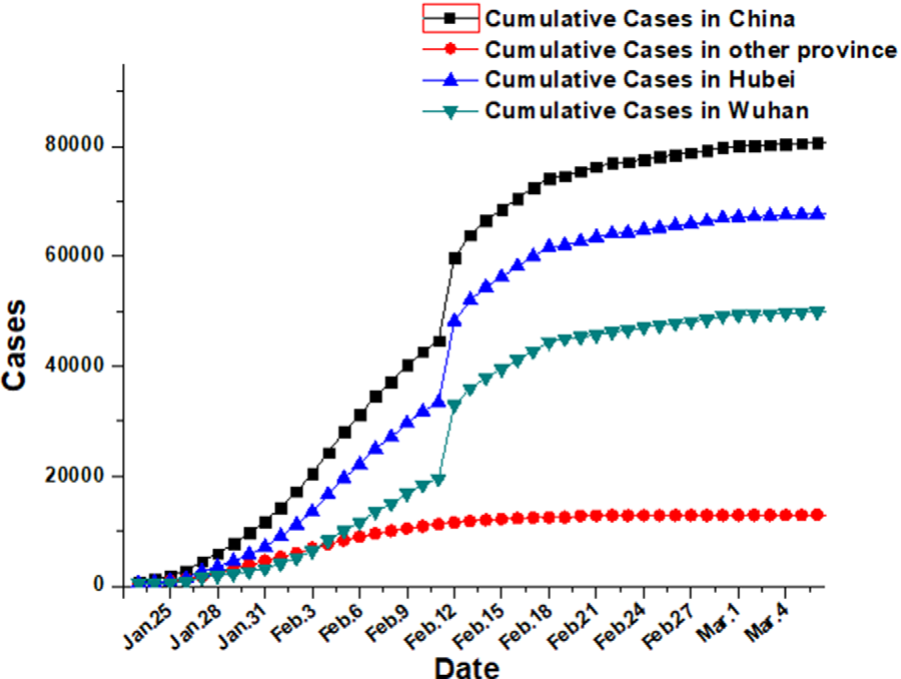
Trend of Confirmed Diagnosis of the National Epidemic.

In Wuhan, the worst-hit area of the outbreak, the number of confirmed cases reached a record high of 38,020 on February 18th. Since then, the number has fallen for 17 consecutive days, but the number of confirmed cases in Wuhan has still risen to 19,011, or 85.7% of the national total, which is the highest since January 24. Hubei Province, especially Wuhan, is the top priority for national epidemic prevention and control (see Figure 2).

The prevention and control measures in Wuhan and even Hubei Province mainly include the 6 aspects described below.

#### Strict Output Prevention

Take measures to “close the city.” The government closed all exits from Wuhan, controlled the source of the infection, and aimed to keep the epidemic from spreading from Wuhan to other cities (states), from cities to rural areas, and from Hubei to other provinces.

#### Classified Treatment

Four types of personnel are classified and treated intensively.

Confirmed patients: Severe patients are sent to designated hospitals, and mild patients are sent to designated hospitals and other medical institutions.

Suspected patients: They need to continue to be watched at the “hot” clinic. If there are insufficient beds, they must be moved to centralized isolation points.

Patients with febrile fever: These patients cannot be ruled out and must also be isolated and observed, the same as suspected patients. The government should isolate them from suspected patients to prevent cross-infection.

Close contact: The government needs to investigate instances of close contact and refer to the treatment of fever patients to isolate them at home after observation.

#### Strengthening Medical Treatment

**FIGURE 3:**
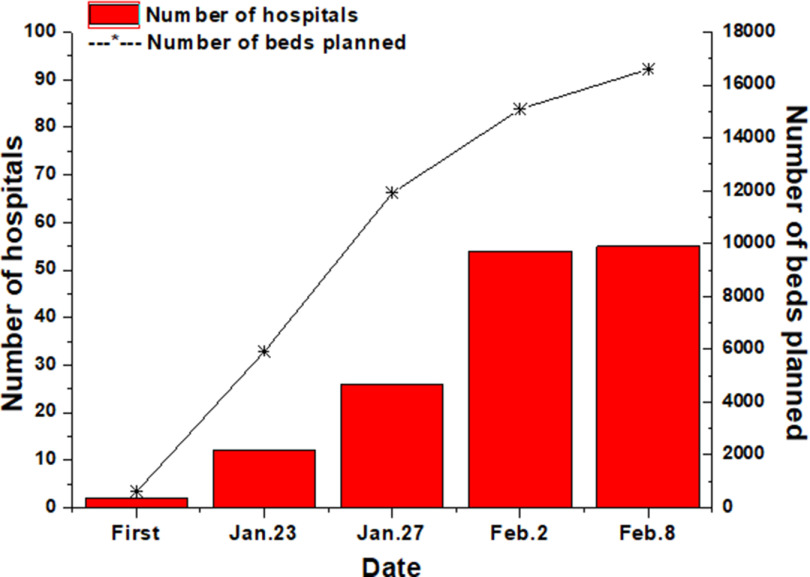
Increasing Beds in Designated Hospitals.

The government continues to increase efforts to treat fevers and diagnose patients. A total of 62 hospitals in Wuhan have set up fever clinics, which can treat 15,000 people during peak hours.^11^ To deal with cross-infection and disease transmission caused by the disordered medical treatment of fever patients, Wuhan has set up designated hospitals in batches (see Figure 3). With the rapid increase in confirmed patients, designated hospitals only treat confirmed severe cases, critical cases, and suspected critical cases.

Huoshenshan Hospital and Leishenshan Hospital were successively built in 10 days. These hospitals have set up auxiliary departments such as infection control, inspection, and radiological diagnosis for the treatment of critical and severe patients.

Since the emergency response, all medical personnel in Hubei Province have been on duty, and more than 80,000 medical personnel in 131 designated hospitals have been engaged in treatment. Preliminary estimates indicate that the number of first-line medical personnel engaged in treatment in the province has exceeded 170,000.^12^

Building “square cabin hospitals”: Mild patients have greater mobility and posed a greater threat of causing infections in the society. Most of these patients are managed only by oral medicine and temperature measurement. The “square cabin hospital” can quickly resolve the shortage of beds for isolating and treating patients in a timely manner. At present, there are 16 “square cabin hospitals” in Wuhan with a total of approximately 13,000 beds to provide diagnosis and treatment for mild patients.

With the expansion of designated hospitals, the diversion of “square cabin hospitals,” and the continuous rise in cure rates, the empty bed rate of designated hospitals has increased significantly. Wuhan has basically realized “beds being ready for patients” since February 21. The rate of empty beds in designated hospitals has exceeded 10% for the first time. The beds have been fully supplied, which can completely meet the requirements of receivables, and quarantines.

Constantly enrich the medical aid of other provinces: On February 6, the initial statistical gap of medical personnel in Hubei Province was approximately 2250. The government actively deploys and uses various resources to maximize the rate of transshipment treatment. As of March 7, a total of 42,600 medical team members had been dispatched to carry out medical treatment in Hubei Province.

**FIGURE 4:**
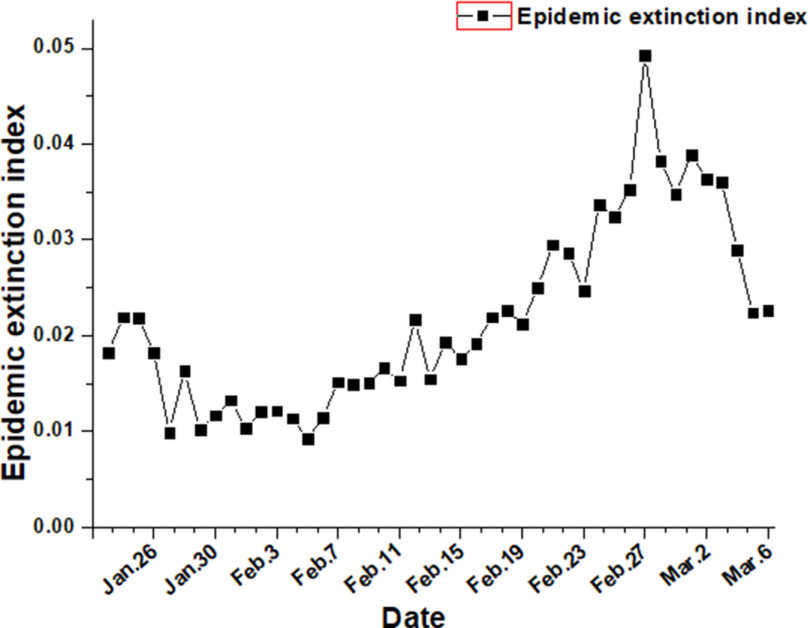
Trend Graph of Epidemic Extinction Index of Hubei.

The epidemic extinction index is the rate of increase in the number of new cures and deaths on a given day compared with the number of existing cases on the previous day. The larger the index, the faster the rate of epidemic extinction. The Hubei epidemic extinction index showed an overall upward trend before February 27, indicating that the use of hospitals and the full input of medical staff have led to a rapid increase in the number of patients cured and discharged (see Figure 4).

#### Addition of Centralized Isolation Points

There are many close-contact cases who need to go to the isolation point for centralized isolation or home isolation. Wuhan had set up approximately 140 centralized isolation points with 14,000 beds.^13^ The construction of various districts is also being stepped up. The isolation points are created by collecting private hospitals, hotels, schools, and other places.

#### Detection of All Suspected Cases

At present, there are approximately 100 nucleic acid testing institutions in Hubei Province, with a daily detection volume of 10,000 copies.^14^ Efficient testing procedures enable more suspected cases to be screened more quickly, which is conducive to the timely treatment of patients.

Nucleic acid tests were performed on cases treated in hospitals and suspected cases observed in isolation. For clinically highly suspected cases, we usually need to repeatedly collect respiratory specimens and use 2 or more reagents to test and verify, or perform virus-specific antibody immunoglobulin (Ig) M and IgG tests to make a comprehensive diagnosis. Due to the long waiting time of nucleic acid detection and the limitation of sampling timing, it is generally not used for infection screening of the general population alone. Nucleic acid detection has the advantages of early, sensitive, high specificity, and easy operation, but the accuracy of nucleic acid detection results is related to the infection time of patients and other factors, such as sample collection, reagent quality, and experimental detection.

#### Community-by-Person Investigation

**FIGURE 5:**
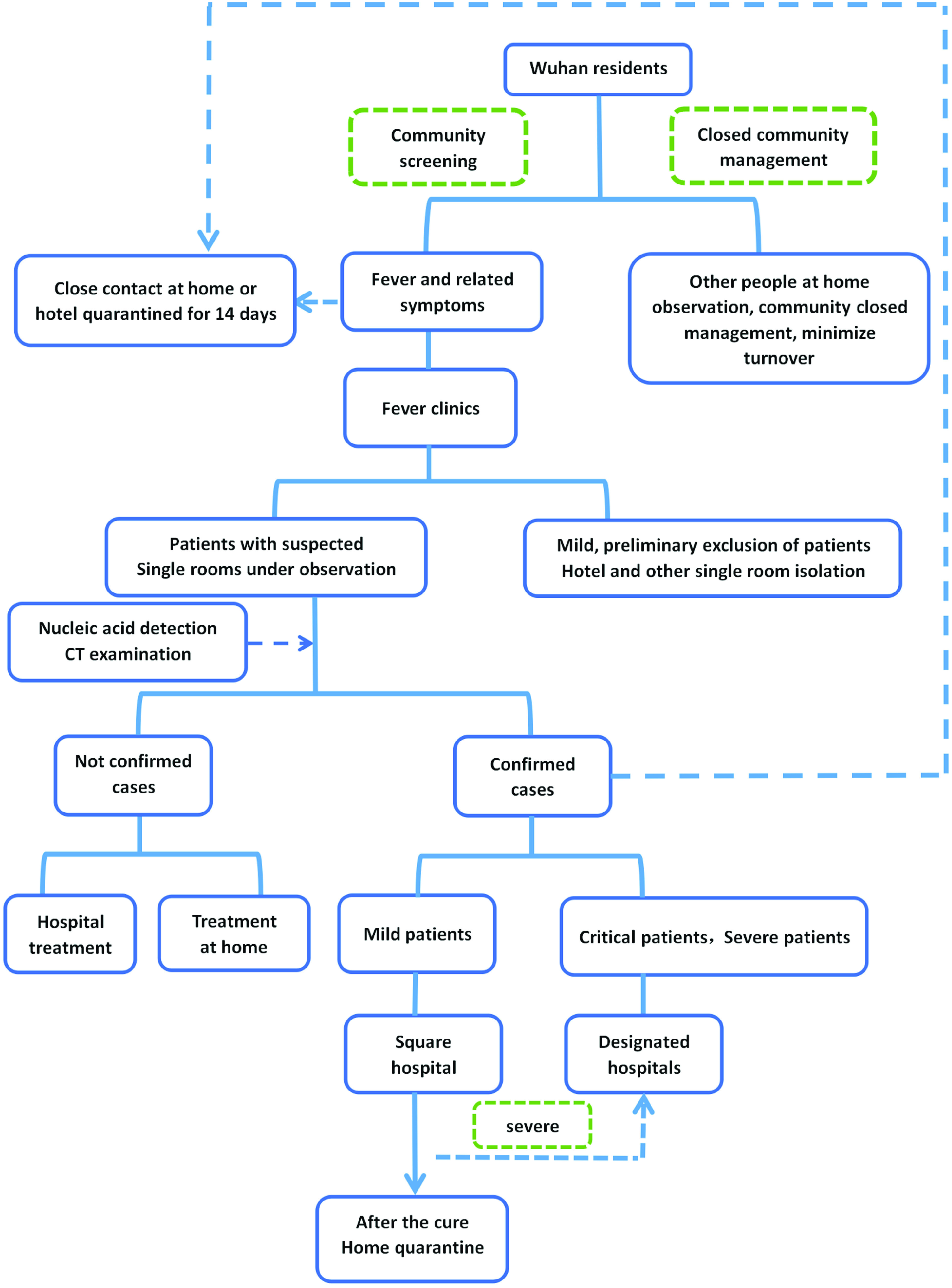
Schematic of Screening and Disposal of Residents.

On February 6, the staffs of Wuhan’s communities carried out blanket surveys of “4 Types of Personnel,” taking temperature measurements, asking for close-contact history, and conducting centralized treatment and isolation (Figure 5). The purpose was to effectively control the source of infection and cut off means of transmission to curb the spread of the epidemic. As of February 9, Wuhan had inspected 4.21 million households for a total of 10.59 million people. The percentage of inspected households in Wuhan reached 98.6% and the number of inspected people 99%; 1499 patients diagnosed with severe illnesses were admitted to hospitals.^15^

### Emergency Management Measures in Provinces Outside Hubei

As the joint prevention and control mechanism commanders, provincial (municipal) party committee secretaries or provincial (municipal) mayors set up an epidemic prevention and control working group. It is composed of a comprehensive group, an epidemic prevention and control group, a medical treatment group, a transportation joint inspection group, a publicity information team, a material security team, a supervision team, and a social stability team. The professional posts, emergency posts, and medical staff of each unit are on standby. According to the data, which are affected by the Chinese Spring Festival and the epidemic, in the early “closed city” period, more than 5 million people left Wuhan.^16^ Epidemic prevention tasks in other provinces outside Hubei have proven difficult as well. The government took the following measures:

External defense input: The government established quarantine stations at highways, bus stations, and airports to register and measure the temperature of passing vehicle personnel, identify those with fever symptoms, and transfer them to designated places for medical observation. The government took the community (village) as the unit, organized staff to go house to house to gather statistics, conducted health tracking of returnees at the place where the epidemic occurred, and promoted prevention and control knowledge.

Internal nonproliferation: Governments have halted crowd-gathering activities, implemented “traveling wearing masks” in public places, and strengthened the inspection of farmers’ markets and the catering industry. They have improved hygiene and investigated and managed the sale of wild animals. The hospital has implemented a precheck and triage system to open green referral channels to patients to protect medical staff, and reserve protective materials.

**FIGURE 6:**
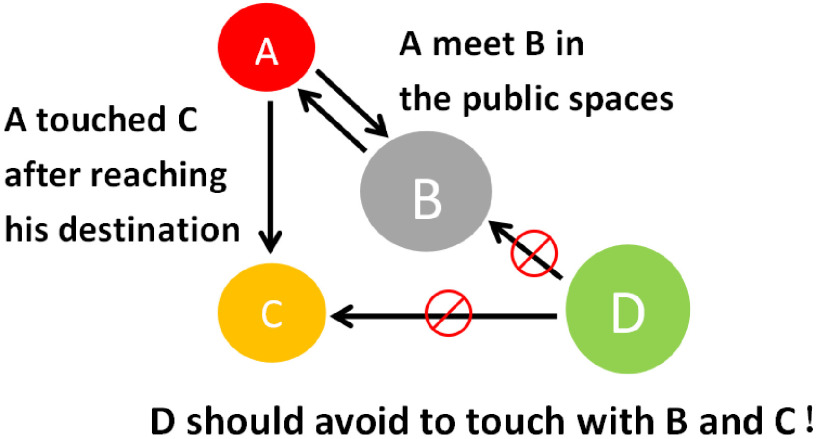
Schematic Diagram of the Four Groups of People.

The focus of epidemic prevention in provinces outside Hubei lies in the investigation of population groups A, B, C, and D, as shown in Figure 6 and Table 1.

**Table 1:** Four types of population screening programs

Personnel Category	Category Description	Troubleshooting Plan
A	1. Coming from Wuhan 2. Those who have recently visited Wuhan in January	The community (village) is used as the unit to investigate the personnel exported from Hubei province, especially Wuhan, and it is required to take the initiative to register with the community (village) health institution, receive regular medical examinations, and actively isolate itself for 14 days. Persons with suspicious symptoms should promptly notify the local health department for screening and screening.
B	People who have contacted A but do not know it (A and B do not know of each other’s existence)	The government publishes the trajectory of confirmed cases in real time. Using big mobile data and the case trajectory applet query function, it is possible to query places where confirmed cases stay online, including flights or railway trips, residential real estate, streets, and shopping malls (supermarkets). Such personnel need to isolate themselves and observe their health status and their family members and contact the CDC of the county (city) where they are located. If there are related suspected symptoms, they should immediately seek medical treatment.
C	Close contacts, family members, friends, colleagues, neighbors	Based on person A, you can find person C, and the district disease control center or community health service center will follow-up with the houses for medical observation.
D	Normal person	Category D personnel reduce going out if conditions permit; reduce contact with As, Cs, or Bs with potential infection; and reduce the possibility of being infected.

## FUTURE DIFFICULTIES AND CHALLENGES

### Hubei Province and Wuhan Still Face Huge Challenges for Future Epidemic Prevention and Control

As of 24:00 on February 8, the epidemic situation in Wuhan was very serious, with a total of 14,982 confirmed cases, accounting for 44.40% of the total confirmed cases in the early stages of the epidemic in China. There were more than 16,594 hospital beds in Wuhan. Given the intensified investigation of the 4 types of personnel and the current growth rate of daily diagnoses and suspected cases in Wuhan, more confirmed and suspected cases will certainly appear in the future, and the number of people requiring treatment and isolation will be greatly increased. Hence, future pressure for intensive treatment is very high. Add that to the shortage of approximately 2250 medical staff across Hubei. Thus, the question arises of how to overcome the pressure of treating diagnosed patients while ensuring that patients with other diseases in the hospital are effectively treated. This situation poses great challenges for the prevention and control of the epidemic in Hubei Province and Wuhan.

### Logistical Support for Epidemic Prevention and Control Will Continue to Face Challenges

As of February 8, Wuhan had recruited 29 designated hospitals for use, and a total of 100,000 medical workers had participated in prevention and control work in the early stages of the epidemic. The number will expand to 55 designated hospitals in the future. In addition, more than 11,000 medical personnel from all over China have been supporting Hubei. Given the presence of so many medical workers, effective logistical support will pose a major challenge for Hubei Province and Wuhan. At the same time, the demand for materials, such as protective clothing and masks, is very prominent.^17^ The demand in Hubei Province is roughly 100,000 pieces of protective medical apparel per day. However, there are only 40 companies with production capacity licenses that meet Chinese standards, and their total production capacity is only 30,000 units per day.^18^ The total number of masks needed every day in China is 21.6 million pieces. Even if China’s maximum production capacity is restored, the daily output is approximately 20 million pieces. Thus, there was still a big gap in February. However, this situation has now improved.

### The End of the Chinese Spring Festival, Resumption of Work, Start of School, Migration of Large Numbers of People, and Population Gatherings Will Challenge Prevention and Control

After the Chinese Spring Festival holiday, many people will be taking return trips. Many companies are currently delaying work in large areas. Schools across the country have taken measures to delay opening. According to data from the Ministry of Transport of China, from January 10 to February 5, 2020, a total of 1.318 billion passengers traveled across the country. It is expected that the number of passengers will be around 400 million during the second half of the Chinese Spring Festival. This is still a large-scale migration group. After the start of school, there were approximately 276 million students and 16.7285 million teachers engaged in all types of academic activity at all levels. At this time, schools become some of the most densely populated places in China. Community-based prevention and control is thus becoming more difficult. After the Spring Festival, people in the community will move more frequently, which will increase the risk of virus transmission. At the same time, because China has a large population and high population density, community work will become more difficult. Furthermore, China is a large agricultural country, with a total rural population of approximately 700 million people, accounting for 50.32% of the total population; medical conditions are worse in rural areas than in cities and should, therefore, be a focus of prevention.

### The Government Should Engage in International Cooperation to Stop the Spread of the Epidemic

Although the WHO has declared the novel coronavirus epidemic a PHEIC, it also acknowledges China’s prevention and control efforts and does not suggest that other countries should impose any travel or trade restrictions. China has mobilized and adopted strong, strict prevention and control measures, notified the WHO and the international community in a timely manner, and shared all virus-related data. The international community should, therefore, show a strong sense of solidarity and provide support for China and other countries that may suffer from the epidemic. Japan, Russia, and other countries have already aided China. A clinical trial of the US antiviral drug remdesivir has been initiated at Wuhan Jinyintan Hospital.^19^ Countries around the world should increase efforts to work together to prevent and control the epidemic and maintain global public health security in accordance with the International Health Regulations and recommendations of the WHO.

### The Need for Psychological Intervention by All Parties in Society Will Increase

The novel coronavirus epidemic began in December 2019 and has characteristics such as unpredictability, threat to life, and uncontrollability. In China, especially Hubei Province, patients and residents have had stress reactions to the epidemic. Such reactions have manifested as fear, anxiety, sensitivity, irritability, depression, disappointment, and anger.^20^ Some high-intensity and long-term medical staff are always in close contact with patients, which can give rise to feelings of fear, tension, and disappointment.^21^ The government should recognize the great need for psychological interventions for the public in the future. In view of the severe situation of the global outbreak, WHO points out that COVID-19 has the characteristics of a pandemic, which is the first pandemic caused by coronavirus and the first pandemic under control, and calls on all governments to make the containment of the epidemic a top priority. The current COVID-19 epidemic has been effectively controlled in China. We hope that China’s experience can provide reference for the epidemic prevention and control work in other countries.
